# Gene expression analysis of aberrant signaling pathways in meningiomas

**DOI:** 10.3892/ol.2013.1363

**Published:** 2013-05-23

**Authors:** MIGUEL TORRES-MARTÍN, VICTOR MARTINEZ-GLEZ, CAROLINA PEÑA-GRANERO, ALBERTO ISLA, LUIS LASSALETTA, JOSE M. DE CAMPOS, GIOVANNY R. PINTO, ROMMEL R. BURBANO, BÁRBARA MELÉNDEZ, JAVIER S. CASTRESANA, JUAN A. REY

**Affiliations:** 1 Neuro-Oncogenetics Laboratory, Research Unit, Madrid, Spain;; 2 INGEMM, Madrid, Spain;; 3 Neurosurgery Department, Madrid, Spain;; 4 Department of Otolaryngology, La Paz University Hospital, IdiPAZ, Madrid, Spain;; 5 Fundación Jiménez Díaz, Madrid, Spain;; 6 Genetics and Molecular Biology Laboratory, Federal University of Piauí, Parnaibá, Brazil;; 7 Human Cytogenetics Laboratory, Federal University of Pará, Belem, Brazil;; 8 Molecular Pathology Research Unit, Virgen de la Salud Hospital, Toledo;; 9 Brain Tumor Biology Unit, University of Navarra, Pamplona, Spain

**Keywords:** meningioma, schwannoma, neurofibromin gene, gene expression arrays, signaling pathways

## Abstract

Examining aberrant pathway alterations is one method for understanding the abnormal signals that are involved in tumorigenesis and tumor progression. In the present study, expression arrays were performed on tumor-related genes in meningiomas. The GE Array Q Series HS-006 was used to determine the expression levels of 96 genes that corresponded to six primary biological regulatory pathways in a series of 42 meningiomas, including 32 grade I, four recurrent grade I and six grade II tumors, in addition to three normal tissue controls. Results showed that 25 genes that were primarily associated with apoptosis and angiogenesis functions were downregulated and 13 genes frequently involving DNA damage repair functions were upregulated. In addition to the inactivation of the neurofibromin gene, *NF2*, which is considered to be an early step in tumorigenesis, variations of other biological regulatory pathways may play a significant role in the development of meningioma.

## Introduction

Meningiomas account for ∼30% of all central nervous system neoplasms and are derived from the arachnoid cells covering the brain. The majority of meningiomas are slow-growing and correspond to grade I tumors, according to the World Health Organization (WHO) classification. Grade II (atypical) and grade III (anaplastic) meningiomas are considerably more aggressive (1). Meningiomas may present as sporadic solitary tumors. In the context of neurofibromatosis type 2 disease (NF2), the tumors may also be characterized by the presence of bilateral acoustic schwannomas (1,2). Molecular genetic alterations that are linked to meningioma tumorigenesis indicate the inactivation of at least one member of the 4.1 super-family of genes, primarily involving the neurofibromin gene, *NF2,* that is located at 22q12.2 or the 4.1B (*DAL-1*) gene, located at 18p11.32 (1,2). Chromosomal losses at other genomic regions, including 1p, 6q, 9p, 10, 14q and 18q, as well as gains in multiple regions, including 1q, are frequently identified in grade II and III tumors (1,3–5). Accordingly, genetic alterations in those specific genomic regions are likely to be associated with the progression of meningioma. Controversial data have been obtained from a low sequence mutation rate that has been identified by the analysis of potential target genes in these locations (1,6–7). The low mutation rate may be due to alternative gene silencing mechanisms, since aberrant promoter hypermethylation of the CpG islands may contribute to changes in the expression status of those genes (8–11). The loss of tumor suppressor genes, including *CDKN2A* and *CDKN2B*, and the involvement of *MEG3, NDRG2, TSLC1, LMO4,* osteopontin and *LEPR,* have been proposed as candidates for progression or recurrence markers in meningiomas (1,12–13). Transcriptomic expression profiling studies using array analyses have been performed in meningiomas (14) and certain meningioma-specific genes or those associated with the WHO grades have been identified.

Recently, common genes that are responsible for the recurrence and progression of meningiomas, or for the existence of differential expression patterns among spinal and intracranial meningiomas, have been identified (15,16). Analyses of low-density microarrays have previously identified two expression subgroups in meningiomas. The tumors exhibited abnormal patterns with deletions at 1p and 14q, in addition to a 22q deletion/*NF2* inactivation (17,18). Attention has been focused on the aberrant signaling pathways in meningiomas. The findings confirm that the most likely implication involves the loss of *NF2*. A number of growth factors and cytokines, and the deregulation of the calcium signaling system, have been proposed to be involved in the development and progression of meningiomas, but no firm conclusions are available (19). This study presents additional data to those that have been previously reported (17,18) with regard to the expression variations of 96 tumor-related genes included in six key signaling pathways in meningiomas. In order to obtain new findings, the present study focused on the signaling pathways that may be altered in these brain tumors. The data show that the pathways that are involved in cell cycle control, DNA damage repair, apoptosis and angiogenesis were more commonly deregulated.

## Materials and methods

The patterns of gene expression levels were analyzed in 42 meningiomas, including 32 grade I, four recurrent grade I and six grade II tumors, using the GE Array Q Series HS-006 (SuperArray, Bethesda, MD, USA) for the analysis of 96 genes corresponding to various biological pathways that are frequently deregulated in tumorigenesis. Group 1 was associated with cell cycle control and DNA damage repair (15 genes), group 2 with apoptosis and cell senescence (14 genes), group 3 with signal transduction molecules and transcription factors (17 genes), group 4 with adhesion (15 genes), group 5 with angiogenesis (22 genes) and group 6 with invasion and metastasis (13 genes).

Detailed information with regard to specific tumor-related genes, housekeeping genes and negative controls is available at http://www.superarray.com and http://www.sabiosciences.com. A control comprising three commercial human adult normal (non-tumoral) RNA from cerebral meninges (U.S. Biological Biochain, Hayward, CA, USA) was used. The manufacturer's instructions were used with small variations, as described (18). Previous array experiment information and data are available at ArrayExpresss (http://www.ebi.ac.uk/arrayexpress). Quantitative polymerase chain reactions (qPCR) were used to validate the microarray data and statistical analyses were performed as previously described (18). A total of four genes (*BCL2L1, FOS, MDM2* and *TIMP1*) that were included in the microarray were selected for the analysis and *GAPDH* was used as an endogenous control gene. Expression data from the arrays and q-PCR were adjusted to a normal distribution to compare the sets of data and expression results were consistent between the two methodologies. A particular gene was considered to be overexpressed if the expression levels were ≥5 times the values obtained in the control samples. A gene was considered to be underexpressed if the expression levels were ≤0.2 of the mean values found in the non-tumoral control samples.

## Results and Discussion

**Figure 1.**
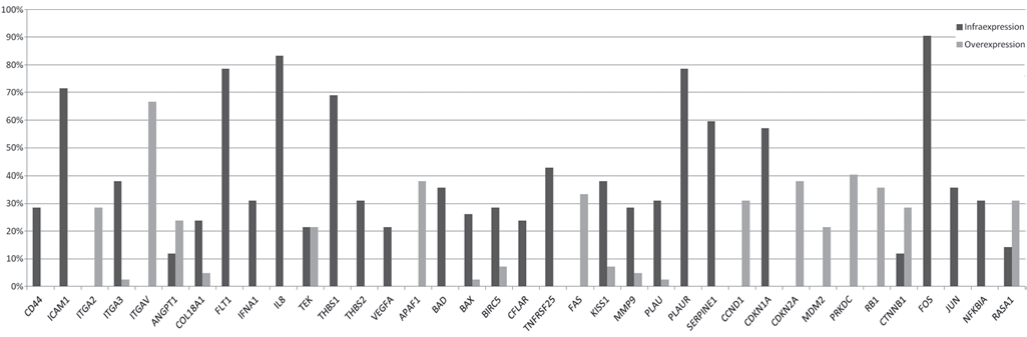
Data obtained from meningiomas with at least 20% deregulation. Genes are named using the official symbols.

A total of 25 of the 96 genes were downregulated in at least 20% of samples. These genes correspond to: CDKN1A (gene group 1); *BAD, BAX, BIRC5, CFLAR* and *TNFRSF25* (group 2); *FOS, JUN* and *NFKBIA* (group 3); *CD44, ICAM1* and *ITGA3* (group 4); *COL18A1, FLT1, IFNA1, IL8, TEK, THBS1, THBS2* and *VEGFA* (group 5); and *KISS1, MMP9, PLAU, PLAUR* and *SERPINE1* (group 6). An additional 56 genes were downregulated in <20% of samples and 15 genes presented no underexpression. However, 14 of these 15 genes were upregulated at variable levels and one remained constant. In 13 genes, the expression values were consistent with the criteria for upregulation in at least 20% of samples, including those of grade I and II. These genes were distributed as follows: *CCND1, CDKN2A, MDM2, PRKDC* and *RB1* (group 1); *APAF1* and *FAS* (group 2); *CTNNB1* and *RAS1* (group 3); *ITGA2* and *ITGAV* (group 4); and *ANGPT1* and *TEK* (group 5). No genes from group 6 (invasion and metastasis) were observed to be overexpressed, which is consistent with the characteristic low-grade malignancy (WHO grade I) of the majority of meningiomas that generally do not exhibit metastasis. A total of 29 genes presented no overexpression, but 28 were underexpressed in at least one sample and involved grade I and II tumors. Of all the meningiomas analyzed, 54 genes were upregulated in <9 samples (<20%). A summary of the main findings is shown in Table I and Fig 1.

The genes that were included in group 2 (apoptosis and cell senescence) were the most frequently altered in the present meningioma study, as 7 out of 14 (50%) genes were abnormally expressed, of which five were found to be downregulated and two upregulated. Accordingly, the low expression levels of the five genes in this functional group may be involved in meningioma development. However, it is difficult to understand the role that is played by the upregulation of the remaining two genes, *APAF1* and *FAS*. Alterations of at least 40% of genes exhibiting significant expression variation, according to the criteria for under- or overexpression present in at least 20% of samples, were identified in two additional functional groups: 10 of 22 (45%) genes in group 5 (angiogenesis) and six of 15 (40%) genes associated with cell cycle control and DNA damage repair (group 1). Although the genes in group 5 were downregulated, those included in group 1 appeared to be overexpressed. Due to the small number of genes that were analyzed, internet databases such as DAVID (http://david.abcc.ncifcrf.gov/) did not show any enriched pathways or gene grouping. Thus, the findings suggest that the alteration of regulatory pathways involved in apoptosis, angiogenesis and DNA repair may be involved in meningioma development as alterations that are secondary to *NF2* inactivation, or in general to the aberrant signaling pathways involving membrane-associated 4.1 family proteins. Not all the deregulated genes mapped to the chromosomal regions are frequently altered in meningiomas.

The proportional gene loss, which may be a result of underexpression due to hemizygosity, in the genomic regions that were frequently deleted (22q, 14q, 1p, 6q, 9p, 10, 18q), may contribute to the deregulation of other genes that are located in surrounding genomic regions. An aberrant gene expression pattern associated with allelic losses at 1p and 14q has been described in meningiomas and the majority of the identified deregulated genes are located outside those genomic regions (18). Moreover, although no statistical analysis was performed due to the small number of cases considered as recurrent (four tumors) or grade II (six cases) in the present study, the more aggressive meningiomas and the grade I tumors were associated with an abnormal cDNA expression pattern, indicating that a subgroup of grade I meningiomas may exhibit a predisposition to evolve into more aggressive forms. The genes that were found to be deregulated concur with those that have been identified in previous studies (14). The aberrant signaling pathway that is predominantly involved in meningioma tumorigenesis thus far identified involves the loss of chromosome 22 and therefore *NF2* gene inactivation (19). Less frequently, cellular signal transduction pathways, including Hedgehog, MAPK, PI3K and Notch, growth factors and cytokines, or calcium signaling pathways also exhibit abnormal deregulation (19). The present data suggest that apoptosis, angiogenesis, cell cycle control and DNA damage repair pathways are commonly altered in meningiomas.

Due to the close molecular genetic association (*NF2* inactivation) between meningioma and schwannoma, the results from the present study were compared with those that were previously reported using the same methods in a schwannoma series (20), in order to identify genes that were equally or differentially expressed in the two entities (Tables II and III). The most frequently altered functional groups differ between the tumor types. The genes that code for proteins involved in apoptosis and cell senescence (group 2) were found to be deregulated in up to 50% of meningioma samples compared with the genes from group 4 (adhesion proteins) that were abnormally regulated in 53% of schwannoma samples). In addition, a concurrent expression alteration of genes included in groups 1 (up to 40%) and 5 (45%) was identified in the meningioma and schwannoma samples. These groups of genes correspond to cell cycle control and DNA damage repair (group 1) and angiogenesis (group 5). With regard to the involvement of specific genes, certain inter-tumoral group variations were identified, including *BRC5, JUN* and *SERPINE1*, which were underexpressed in >30% of meningiomas compared with <10% of schwannomas. Low *EGFR* levels were identified in up to 40% of schwannomas but <10% of meningiomas. *PIK3R1, ITGA4* and *ITGA6* were overexpressed in >35% of schwannomas versus <10% of meningiomas. By contrast, *CTNB1* was upregulated in the majority of meningiomas but only in a few schwannoma tumors. Significant differences were noted for three additional genes. *NCAM1* was overexpressed in 30% of schwannomas and downregulated in a few meningioma tumors. *CD44* and *ITGA3* were underexpressed in meningiomas and over-expressed in schwannomas. *FOS, ICAM1* and *FLT1* were underexpressed and *ITGAV* was upregulated in the two tumor types.

The present results thus complete the previous data to classify gene expression in neurogenic neoplasms (17). In addition, the findings are associated with the characterization of genes that are abnormally expressed in meningiomas, as described previously, and are concurrent with 1p and 14q deletions. However, the deregulated genes are not always located in those specific genomic regions (18). The findings from the present study show that apoptosis, angiogenesis, cell cycle control and DNA damage repair pathways are commonly deregulated in meningiomas.

In conclusion, the identification of genes that are abnormally expressed in meningiomas and schwannomas may be useful for determining common therapeutic target strategies, primarily in NF2 patients who frequently present with the two types of neoplasms.

## Figures and Tables

**Table I.** Genes that were derugulated in at least a 20% of samples.

Gene Symbol	Unigene	Localization	Infra-expression (%)	Overexpression (%)	Functional group
*CDKN1A*	Hs.370771	6p21.2	57	0	1
*BAD*	Hs.370254	11q13.1	36	0	2
*BAX*	Hs.624291	19q13.3–q13.4	26	2	2
*BIRC5*	Hs.514527	17q25	29	7	2
*CFLAR*	Hs.390736	2q33–q34	24	0	2
*TNFRSF25*	Hs.462529	1p36.2	43	0	2
*FOS*	Hs.731317	14q24.3	90	0	3
*JUN*	Hs.696684	1p32–p31	36	0	3
*NFKBIA*	Hs.81328	14q13	31	0	3
*CD44*	Hs.502328	11p13	29	0	4
*ICAM1*	Hs.643447	19p13.2	71	0	4
*ITGA3*	Hs.265829	17q21.33	38	2	4
*COL18A1*	Hs.517356	21q22.3	24	5	5
*FLT1*	Hs.594454	13q12	79	0	5
*IFNA1*	Hs.37026	9p22	31	0	5
*IL8*	Hs.624	4q13–q21	83	0	5
*TEK*	Hs.89640	9p21	21	21	5
*THBS1*	Hs.164226	15q15	69	0	5
*THBS2*	Hs.371147	6q27	31	0	5
*VEGFA*	Hs.73793	6p12	21	0	5
*KISS1*	Hs.95008	1q32	38	7	6
*MMP9*	Hs.297413	20q11.2–q13.1	29	5	6
*PLAU*	Hs.77274	10q24	31	2	6
*PLAUR*	Hs.466871	19q13	79	0	6
*SERPINE1*	Hs.414795	7q22.1	60	0	6
*CCND1*	Hs.523852	11q13	0	31	1
*CDKN2A*	Hs.512599	9p21	0	38	1
*MDM2*	Hs.484551	12q14.3–q15	0	21	1
*PRKDC*	Hs.491682	8q11	0	40	1
*RB1*	Hs.408528	13q14.2	0	36	1
*APAF1*	Hs.552567	12q23	0	38	2
*FAS*	Hs.244139	10q24.1	0	33	2
*CTNNB1*	Hs.476018	3p21	12	29	3
*RASA1*	Hs.664080	5q13.3	14	31	3
*ITGA2*	Hs.482077	5q11.2	0	29	4
*ITGAV*	Hs.436873	2q31–q32	0	67	4
*ANGPT1*	Hs.369675	8q23.1	12	24	5
*TEK*^a^	Hs.89640	9p21	21	21	5

Official gene symbols, Unigene database annotations and chromosomal location are shown.

a *TEK* was both upregulated and downregulated.

**Table II.** Genes with differing trends between meningiomas and schwannomas.

Expression	Gene	Meningioma (%)	Schwannoma (%)
Downregulation	*BIRC5*	∼30	<10
	*JUN*	∼30	<10
	*SERPINE1*	∼30	<10
	*EGFR*	<10	40
	*CD44*	28	-
	*ITGA3*	36	-
	*NCAM*	<10	-
Upregulation	*PIK3R1*	<10	>30
	*ITGA4*	<10	>30
	*ITGA6*	<10	>30
	*CTNB1*	28	<10
	*CD44*	-	13
	*ITGA3*	-	30
	*NCAM*	-	30

**Table III.** Genes showing a similar trend between meningiomas and schwannomas.

Expression	Gene	Meningioma (%)	Schwannoma (%)
Downregulation	*FOS*	90	65
	*ICAM1*	71	61
	*FLY1*	78	48
Upregulation	*ITCAV*	66	61
